# Effects of combined metformin and cabergoline versus metformin alone on inflammatory markers in Iraqi patients with PCOS and hyperprolactinemia: a randomized clinical trial

**DOI:** 10.25122/jml-2026-0012

**Published:** 2026-05

**Authors:** Inas Naser Hamad, Sinaa Abdul Amir Kadhim, Mohammad Bassim, Swadi Asma, Fawzi Hayder Adnan

**Affiliations:** 1 Maternity and Pediatric Teaching Hospital, Al Diwaniyah, Iraq; 2 Department of Pharmacology, College of Medicine, University of Al-Qadisiyah, Iraq; 3 Department of Pharmacy, Al-Mustafa University College, Baghdad, Iraq

**Keywords:** Polycystic ovary syndrome, hormones, GnRH, obesity, PCOS, polycystic ovary syndrome, T2DM, type 2 diabetes mellitus, BMI, body mass index, ELFA, enzyme-linked fluorescent immunoassay, US, ultrasound, LH, luteinizing hormone, FSH, follicle-stimulating hormone, PRL, prolactin, TSH, thyroid-stimulating hormone, AMH, anti-Müllerian hormone

## Abstract

Polycystic ovarian syndrome (PCOS) is by far the most prevalent metabolic disease affecting women during their reproductive life. Obesity is a frequently associated manifestation of polycystic ovary syndrome. Central body fat accumulation has been associated with the production of a variety of cytokines associated with low-grade inflammation in polycystic ovary syndrome. This study aimed to evaluate the effect of metformin and cabergoline, each alone and in combination, on several immune markers in women with hyperprolactinemia. The three-arm interventional study included 75 women with PCOS according to the Rotterdam criteria. They were categorized into three groups: those given metformin alone, those given cabergoline alone, and those given combined agents. Baseline and follow-up characteristics included immune markers such as anti-glutamic acid decarboxylase (anti-GAD) antibodies, gonadotropin-releasing hormone (GnRH) antibodies, and interleukin-18 (IL-18). It was observed that the use of either drug alone resulted in no significant change in the level of anti-GAD; however, combined use of the two drugs resulted in a significant reduction in the level, indicating that these two drugs acted synergistically. It was observed that the combined use of either drug resulted in a more significant reduction of GnRH antibody level. It was observed that the combined use of either drug resulted in a more significant reduction of IL-18 level (*P* <0.001). Metformin and cabergoline are efficient, act synergistically, and are safe when used in combination, reducing IL-8 levels and autoantibodies to GAD and GnRH in women with PCOS.

## Introduction

Women with clinical evidence of menstruation abnormalities, elevated testosterone levels, and ovarian cystic alterations are said to have polycystic ovary syndrome (PCOS) [1,2]. This complex clinical condition may be dominated by hyperandrogenemia and, hence, be considered predominantly biochemical, or by polycystic ovaries and, therefore, termed morphological [3]. It has been estimated that approximately 7% of women of reproductive age have the disease [4]. In the United States, the disease is estimated to affect about 5 million women of reproductive age, according to the National Institutes of Health Office of Disease Prevention [5]. Indeed, it is the most prevalent hormonal condition among women between 18 and 45 years of age [6].

PCOS has also been associated with chronic low-grade inflammation [7,8]. Multiple studies have found a link between persistent low-grade inflammation and hyperandrogenism in women with PCOS. Nevertheless, the causal mechanism of this link remains unknown [9]. In women with PCOS, adipose tissue is recognized as an essential source of overproduced pro-inflammatory mediators, leading to chronic inflammation [10]. The nature of the relationship among hyperandrogenism, insulin resistance, and chronic inflammation is not yet fully understood [9]. Contradictory findings have been reported regarding the association between acute-phase reactants and PCOS, including C-reactive protein (CRP), circulating cytokines such as IL-16 and IL-18, and tumor necrosis factor-alpha (TNF-α) [11-14].

Treatment of PCOS includes a variety of pharmacological agents. Metformin is considered an insulin sensitizer (biguanide) [15]. It does not affect insulin secretion but may enhance insulin actions [16]. It is considered the first-choice therapy for type 2 diabetes (T2DM) [17]. It works by decreasing blood lipid levels, decreasing hepatic glucose synthesis, stimulating the liver and skeletal muscles to conduct insulin-mediated glucose uptake, and decreasing the use of gluconeogenic substrates [18]. Metformin, as an insulin-sensitizing drug, may ameliorate the inflammatory state in PCOS, according to preliminary studies; however, the evidence remains equivocal [9].

Cabergoline is a dopamine receptor agonist. Cabergoline is more reactive to dopamine D2 receptors and has a maximum serum half-life of 43 hours. Patients suffering from hyperprolactinemia may thus benefit from the use of this medicine. Few studies have been conducted on the inhibition of prolactin by long-acting dopamine agonists (cabergoline) in patients with PCOS [19].

Cabergoline was administered primarily for the treatment of hyperprolactinemia in women diagnosed with PCOS. Dopamine agonists such as cabergoline are considered first-line therapy for hyperprolactinemia and may improve endocrine and metabolic disturbances associated with elevated prolactin levels.

In addition to its prolactin-lowering effects, emerging evidence suggests that dopamine agonists may influence inflammatory and metabolic pathways. Therefore, evaluating cabergoline in combination with metformin may provide additional insights into potential synergistic anti-inflammatory effects in PCOS.

Previous reports have shown a role for cabergoline in reducing serum pro-inflammatory cytokine levels in patients with hyperprolactinemia, but its effect on inflammation in PCOS is uncertain [20].

The poverty of Iraqi literature addressing the function of metformin and cabergoline in women with PCOS and the inconsistent results from preliminary studies published worldwide with this regard make a clear justification for the planning and conduction of the current study, aiming at exploring the effects of metformin and cabergoline alone or in combination on inflammatory responses in an Iraqi female sample diagnosed with PCOS with hyperprolactinemia.

## Material and methods

### Trial design

**Figure 1:**
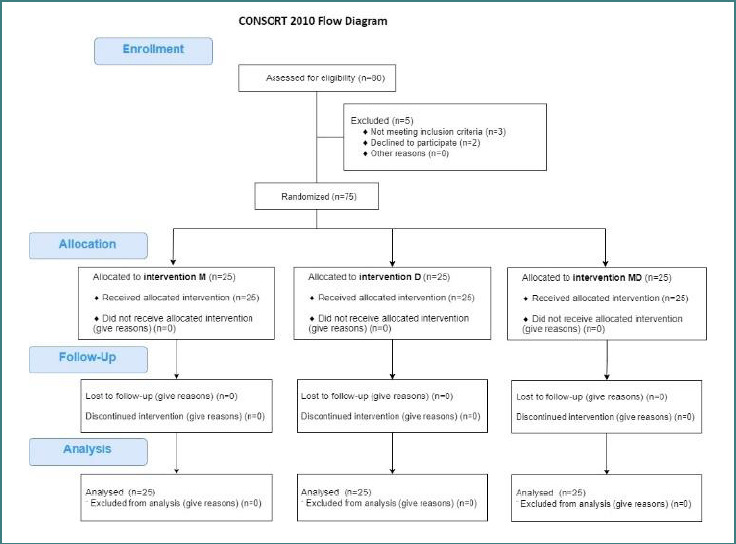
Flow chart of the study

A three-arm, parallel-group, open-label interventional study was conducted involving 75 women with PCOS diagnosed according to the Rotterdam criteria (Rotterdam, 2004) [21]. The diagnosis was made by two specialist gynecologists after detailed history taking, clinical examination, and the required investigations. Patients were then randomized into three groups. Group M included 25 women who received oral metformin 500 mg tablets twice daily for 90 days (Glucophage, Merck Serono, Darmstadt, Germany). Group D included 25 women who received oral cabergoline 0.5 mg once weekly for 90 days (Pergolin, ASIA Pharmaceutical Industries, Aleppo, Syria). Group MD included 25 women who received both metformin and cabergoline for 90 days. Baseline and 90-day follow-up assessments included body mass index and serum inflammatory marker levels, including interleukin-18 (IL-18), anti-glutamic acid decarboxylase antibody (anti-GAD antibody), and gonadotropin-releasing hormone autoantibody (GnRH Ab). Figure 1 illustrates the study flow chart. The study was reported in accordance with the CONSORT 2010 statement. The trial was registered at ClinicalTrials.gov on August 8, 2023, with the identifier NCT05981742.

### Study setting

The patients were recruited from the gynecology outpatient clinic and underwent laboratory testing and ultrasound examinations at the Maternity and Pediatrics Teaching Hospital in Al-Diwaniyah Province, Iraq, during the period from September 21, 2022, to March 31, 2023.

### Inclusion criteria

Female patients aged 18–40 years, diagnosed with PCOS with hyperprolactinemia based on the Rotterdam 2004 criteria [21], with a body mass index (BMI) < 40 kg/m^2^.

### Exclusion criteria

Patients with comorbid conditions, including diabetes mellitus, essential hypertension, and thyroid disease, and patients planning for conception.

### Randomization

Computer-based randomization was used, with participants assigned to three groups. After PCOS diagnosis, patients were numbered and randomized into three groups using the online Research Randomizer software.

### Sample size estimation

Sample size estimation was based on the following equation:

Minimum sample size (*n*) = pX(1-p)XZ^2^/Me^2^

where *n* is the minimum sample size, and p represents the PCOS prevalence, which was reported by Deswal *et al*. [22] to be as high as 20% among women of fertile age. Z represents the Z-score at a 95% confidence interval, which equals 1.96. ME represents the margin of error, which is accepted according to Daniel [23] as 0.05. Thus, the minimum sample size was estimated to be approximately 24.6, and accordingly, the sample size was set at 25 for each group.

### Procedures

BMI calculation was done according to the following equation [24]:

BMI = weight in kg/ (height in m)^2^.

### Laboratory sample preparation

A 10 mL venous blood sample was obtained from each patient. After collection, the blood was allowed to coagulate. The clot was then removed by centrifugation at 2,000–3,000 rpm for 20 minutes.

### Analysis

Laboratory analysis was performed using a double-sandwich ELISA technique (ELISA reader PKL PPC 142, PARAMEDICAL srl, Salerno, Italy), which can determine the concentration of the required materials in human serum. IL-18 was measured using a Human Interleukin-18 (IL-18) ELISA Kit (BT-LAB, Shanghai Korain Biotech Co., Ltd., China). Anti-GAD Ab. was measured using a Human Anti-Glutamic Acid Decarboxylase Antibody (Anti-GAD Antibody) ELISA Kit (BT-LAB, Shanghai Korain Biotech Co., Ltd., China), and GnRH Ab. was measured using a Human Gonadotropin-Releasing Hormone Auto-Antibody (GnRH-A-Ab) ELISA Kit (Shanghai Korain Biotech Co., Ltd., China).

Hyperprolactinemia was confirmed by measuring baseline serum prolactin (PRL) levels using standard laboratory assays. Elevated prolactin levels above the laboratory reference range were considered diagnostic for hyperprolactinemia.

Secondary causes of hyperprolactinemia, including thyroid disease, medication-induced hyperprolactinemia, and other endocrine disorders, were excluded through clinical history and laboratory evaluation.

Patients were diagnosed with PCOS based on the Rotterdam diagnostic criteria, which require at least two of the following: oligo-ovulation or anovulation, clinical or biochemical hyperandrogenism, and polycystic ovarian morphology on ultrasound.

### Trial registration

The trial was registered on ClinicalTrials.gov on August 8, 2023 (Identifier: NCT05981742).

### Statistical analysis

Data were collected, summarized, analyzed, and presented using the Statistical Package for Social Sciences (SPSS), version 23. One-way analysis of variance (ANOVA) was used to evaluate differences in the means of numeric variables among more than two groups, provided that these numeric variables were normally distributed, while the Kruskal-Wallis test was used for non-normally distributed variables. Paired *t*-tests were used to compare means before and after treatment. The level of significance was set at *P* ≤ 0.05.

## Results

Table 1 compares the demographic features of the study groups. No statistically significant difference in mean age was observed among study groups (*P* = 0.149). The mean ages were 24.80 ± 4.87 years, 24.24 ± 4.54 years, and 22.32 ± 4.54 years, respectively, and the age range was 15-39 years. In addition, there were no statistically significant differences in mean BMI among the study groups (*P* = 0.231). The mean BMI values were 29.09 ± 4.49 kg/m^2^, 30.18 ± 4.55 kg/m^2^, and 29.21 ± 2.92 kg/m^2^, respectively, and the BMI range was 18.3-42 kg/m^2^.

**Table 1:** Comparison of demographic characteristics among study groups

Characteristic	Group MD	Group M	Group D	*P* value
Age (years)				
Mean ± SD	24.80 ± 4.87	24.24 ± 4.54	22.32 ± 4.54	0.149
Range	15–39	16–35	16–34	
BMI (kg/m^2^)				
Mean ± SD	29.09 ± 4.49	30.18 ± 4.55	29.21 ± 2.92	0.231
Range	20–37.1	22.26–42	18.3–29.2	

Changes in serum Anti-GAD antibody and serum Anti-GnRH antibody are shown in Table 2. It was observed that using either drug alone did not significantly change anti-GAD antibody levels; however, the combined use of the two drugs resulted in a significant reduction, suggesting a possible synergistic effect. It was also observed that the combined use of the two drugs resulted in a more significant reduction in GnRH antibody levels. Similarly, the combined use of the two drugs resulted in a more significant reduction in IL-18 levels.

**Table 2:** Comparison of immune markers serum levels among study groups

Characteristic	Group MD	Group M	Group D	*P* value
Anti-GAD Ab. before (ng/mL)				
Mean ±SD	7.11 ± 3.58	7.83 ± 4.54	6.78 ± 3.94	0.643
Range	0.38–14.41	1.63–20.89	0.38–13.82	
Anti-GAD Ab. after (ng/mL)				
Mean ±SD	5.05 ± 2.36	8.63 ± 5.19	6.17 ± 2.69	0.003**
Range	0.24–10.16	0.56–22.25	0.71–13.38	
*P*-value	0.033 Pa*	0.599 Pa NS	0.531 Pa NS	
GnRH Ab. before (pg/mL)				
Mean ±SD	860.13 ± 173.86	908.53 ± 381.93	948.18 ± 431.64	0.670
Range	582.72–1417.49	304.78–2010.87	323.98–2269.61	
GnRH Ab. after (pg/mL)				
Mean ±SD	438.51 ± 145.29	524.06 ± 209.00	657.67 ± 123.26	0.182
Range	334.96–1004.22	287.27–1275.77	272.78–823.82	
*P*-value	< 0.001 Pa ***	< 0.001 Pa ***	0.003 Pa **	
IL-18 (pg/mL) Before				
Mean ±SD	294.00 ± 69.42	316.95 ± 39.52	303.89 ± 92.98	0.519
Range	182.07–449.39	242.48–416.13	146.36–484.37	
IL-18 (pg/mL) After				
Mean ±SD	84.61 ± 42.82	126.01 ± 77.98	128.66 ± 59.95	0.023*
Range	10.28–194.20	14.92–382.28	22.75–267.82	
*P*-value	<0.001 Pa ***	<0.001 Pa ***	<0.001 Pa ***	

SD, standard deviation; Pa, Paired t-test; *significant at P ≤ 0.05; *** significant at P ≤ 0.001

## Discussion

According to the current study, no significant variations were found in mean serum IL-18 levels among study groups. Serum IL-18 levels ranged from 146.36 to 484.37 pg/mL, and mean levels from 294.00 to 316.95 pg/mL. Yang *et al*. reported a mean serum IL-18 range of 174.3 to 243.1 pg/mL based on the presence or absence of insulin resistance [25], which is slightly lower than the mean range in our study. Therefore, despite the lack of a control group in the current study, it appears that serum IL-18 is higher in women with PCOS, suggesting a possible pathogenic role. In line with this suggestion, Escobar-Morreale *et al*. found that serum IL-18 was significantly higher in women with PCOS compared with healthy control women [26].

Elevation of IL-18 [26-28] and several other inflammatory markers, including CRP [29,30], TNF-α, and interleukin-6 [31], has also been reported in patients with PCOS. Despite this, the inflammatory nature of PCOS remains controversial because of the wide variation in reported findings; hence, insufficient data exist to definitively resolve this issue [29,32]. According to previous studies, the relationship between PCOS and low-grade inflammation is not entirely conclusive and may need to be examined after clear classification of individuals based on BMI or other factors, using clinically relevant cutoffs [32].

In this study, anti-GAD Ab. levels showed no significant variation among the study groups. The mean level ranged from 3.11 ± 1.58 to 3.83 ± 1.54 ng/mL, while individual levels ranged from 0.38 to 6.89 U/mL. No previous study has measured anti-GAD levels in women with PCOS; however, in a single case report, the level was estimated to be 28.9 IU/mL [33], which was claimed to represent a latent case of type 1 diabetes mellitus. However, in a previous Iraqi study, all cases of PCOS were negative for GAD antibody [34], and we agree with this, as in almost all enrolled cases in the present study, the level was negative (<5 U/mL) [35].

In the present study, administration of either medication alone did not result in a significant change in anti-GAD levels; however, coadministration of the two medications resulted in a significant reduction, indicating that these two drugs may have acted synergistically. Shigiyama *et al*. reported a case in 2016 of a young woman with PCOS who had a high anti-GAD titer (40.0 U/mL; normal range <1.5 U/mL) and was mistakenly treated as having type 2 diabetes mellitus. After anti-GAD positivity was detected, she was treated as having type 1 diabetes mellitus, with an excellent response [36].

Pisklakova *et al*. reported a case of late-onset autoimmune diabetes mellitus in a young female with PCOS who was treated for T2DM with no response. The patient had a glutamic acid decarboxylase (GAD-65) antibody titer greater than 28.9 IU/mL (normal range: 0–5.0 IU/mL). Among patients with PCOS, the risk of T2DM is five to ten times higher than usual; however, no link has been found between PCOS and T2DM, including LADA [33].

In the current study, there was no control group for comparison; however, anti-GAD levels ranged from 0.38 to 20.39 IU/mL, indicating that a significant proportion of women with PCOS enrolled in this study had positive anti-GAD antibodies. Treatment with metformin and cabergoline in combination resulted in a significant reduction in anti-GAD levels. The mechanism of this synergistic activity is not known, but it may be suggested that these two drugs, by reducing body mass, particularly waist circumference and visceral fat, result in a reduced chronic inflammatory response, of which the anti-GAD antibody may be one component.

In this study, the combined use of the two drugs resulted in a greater reduction in GnRH antibody levels. Kem *et al*. evaluated the association between these antibodies and PCOS and found that GnRHR-ECL2-AAb levels were considerably elevated in individuals with PCOS compared with a normal control group. Their presence may have important etiological, diagnostic, and therapeutic implications [37]. Therefore, the use of metformin and cabergoline, either alone or in combination, may reduce these effects by lowering antibody levels.

Sattler *et al.*, using a recombinant fusion protein of full-length human GnRH-R and firefly luciferase, performed an in vitro test to measure autoantibodies against GnRH-R (GnRH-R-aAb). For standardization, a commercial rabbit antiserum against human GnRH-R was used. Serum samples from control individuals and other European PCOS patient cohorts (*n* = 1051) were studied. Contrary to our findings, they concluded that natural GnRH-R-aAb is present in only a very small proportion of adult control individuals and PCOS patients of European descent, and that GnRH-R has not been proven to be a significant autoantigen in PCOS [38].

No prior data are available on the effects of metformin and/or cabergoline on the levels of these antibodies; therefore, further research is needed to validate the current results and elucidate the mechanism by which these antibodies are reduced in response to such treatment. However, it may be suggested that both drugs, by reducing visceral fat, subsequently reduce the low-grade inflammatory response through decreased cytokine production, thereby reducing the production of these antibodies. This proposed mechanism needs further experimental and in vitro studies to be clarified.

Concerning IL-18, we demonstrated that metformin alone significantly reduced serum IL-18, and similarly, cabergoline resulted in a significant reduction in serum IL-18. Additionally, the combination of the two drugs resulted in a greater reduction in serum IL-18 levels, suggesting a possible synergistic effect when used simultaneously. Elevated serum IL-18 levels in women with PCOS have been shown previously [39], and this elevation was linked to overweight and obesity, suggesting that central adipose tissue is associated with low-grade inflammation in women with PCOS. In one recent Iraqi study, the authors showed that serum IL-18 is elevated even in lean women with PCOS [40]. Thus, our finding that IL-18 was reduced may greatly contribute to reducing the impact of the inflammatory response in women with PCOS and may therefore improve many clinical issues associated with this disease, such as infertility and menstrual irregularities.

The inclusion of anti-GAD and anti-GnRH antibodies was intended to explore potential immune-endocrine mechanisms that may contribute to the pathophysiology of PCOS. Growing evidence suggests that immune dysregulation and low-grade inflammation may contribute to the syndrome.

IL-18 was selected as a principal inflammatory marker because it is a pro-inflammatory cytokine that has been reported to be elevated in women with PCOS and associated with insulin resistance and adiposity.

Al-Qadhi *et al*. demonstrated that metformin treatment, at the same dose and duration as in our study, markedly and significantly reduced serum IL-18 levels in women with PCOS; thus, our study is consistent with their findings [41]. More recently, Alzamily *et al*. also found that metformin can significantly lower serum IL-18 levels in individuals with PCOS, further validating our findings [42]. Another study by El Mekkawi *et al*. reported reduced IL-6 and IL-18 levels in metformin-treated PCOS patients [43]. According to previous studies, metformin inhibits nuclear factor-κB (NF-κB), thereby decreasing the inflammatory response, as NF-κB is a protein that activates genes encoding cytokines, through AMP-activated protein kinase (AMPK)-dependent and AMPK-independent pathways [44].

To the best of our knowledge, this is the first study to demonstrate that cabergoline reduces serum IL-18 levels in women with PCOS. Further research is needed to validate our results and disclose the exact mechanism of action of cabergoline in reducing IL-18 in women with PCOS. However, it should be emphasized that the combined use of cabergoline and metformin exhibited a synergistic effect through a mechanism that remains incompletely understood.

### Limitations

Several limitations were observed in this study. First, the short duration of the study and second, the open-label nature of the study may introduce bias compared with double-blinded trials. In addition to the study’s relatively short duration, the present investigation focused primarily on laboratory inflammatory markers rather than on clinical outcomes such as ovulation rate, menstrual regularity, or metabolic changes. Future studies should include these clinically relevant outcomes.

Metformin and cabergoline are effective and safe and may act synergistically when used together to reduce IL-18 levels and autoantibodies against GAD and GnRH in women with PCOS.

## Data Availability

The underlying data are available from the corresponding author upon reasonable request.
